# Early immune and host cell responses to *Cryptosporidium* infection

**DOI:** 10.3389/fpara.2023.1113950

**Published:** 2023-01-27

**Authors:** Jan R. Mead

**Affiliations:** 1 Department of Pediatrics, Children’s Healthcare Organization of Atlanta, Emory University, Atlanta, GA, United States,; 2 Atlanta Veterans Affairs Medical Center, Decatur, GA, United States

**Keywords:** *Cryptosporidium*, innate immunity, dendritic cells, interferon, epithelial cells, TLRs, inflammasomes, cytokines

## Abstract

*Cryptosporidium* spp. are opportunistic protozoan parasites that infect epithelial cells of the small intestine and cause diarrheal illness in both immunocompetent and immunodeficient individuals. These infections may be more severe in immunocompromised individuals and young children, especially in children under 2 in developing countries. The parasite has a global distribution and is an important cause of childhood diarrhea where it may result in cognitive impairment and growth deficits. Current therapies are limited with nitazoxanide being the only FDA-approved drug. However, it is not efficacious in immunocompromised patients. Additionally, there are no vaccines for cryptosporidiosis available. While acquired immunity is needed to clear *Cryptosporidium* parasites completely, innate immunity and early responses to infection are important in keeping the infection in check so that adaptive responses have time to develop. Infection is localized to the epithelial cells of the gut. Therefore, host cell defenses are important in the early response to infection and may be triggered through toll receptors or inflammasomes which induce a number of signal pathways, interferons, cytokines, and other immune mediators. Chemokines and chemokine receptors are upregulated which recruit immune cells such neutrophils, NK cells, and macrophages to the infection site to help in host cell defense as well as dendritic cells that are an important bridge between innate and adaptive responses. This review will focus on the host cell responses and the immune responses that are important in the early stages of infection.

## Introduction

*Cryptosporidium* spp. are opportunistic protozoan parasites that infect epithelial cells of the small intestine and cause diarrheal illness in both immunocompetent and immunodeficient individuals. Clinical signs vary considerably depending on the immunologic status of the host. In immunocompetent individuals, symptoms may range from asymptomatic to more severe signs of illness which may include a clinical presentation of watery diarrhea, abdominal pain (cramping), nausea, vomiting, anorexia, and mild fever. Typically, clinical signs abate in 1–2 weeks. In immunocompromised individuals such as AIDS patients, transplant recipients, and individuals undergoing chemotherapy, the disease can be severe and even fatal (Checkley et al., 2015). Infections may be protracted, and possibly involve extraintestinal sites of infection including the gall bladder, hepatobiliary ducts, pancreatic ducts, and respiratory tract (Shikani and Weiss, 2014).

The majority of human infections appear to be caused by two species: *Cryptosporidium parvum* and *Cryptosporidium hominis*. Most experimental studies relevant to humans have focused on the zoonotic species *C. parvum* which infects a number of animals as well as humans, including various immunocompromised and neonatal mice while adult wild-type mice are essentially refractory to infection. To a far lesser extent, studies have been performed with *C. hominis*, primarily due to limitations propagating the species (passage of *C. hominis* is accomplished in gnotobiotic pigs). Experimental work has also been performed with the murine species *C. muris* (McDonald et al., 1992) and *C. tyzzeri* (Ren et al., 2011; Sateriale et al., 2019). These species of *Cryptosporidium* are naturally found in mice, with a host range restricted mainly to rodents and are not considered human pathogens. The latter species has the advantage of primarily infecting the intestinal tract and for which a genetically modified traceable strain has been developed (Sateriale et al., 2019).

Two important immune mediators in response and recovery from cryptosporidiosis are IFN-γ production and CD4^+^ cells (Flanigan, 1994; Gomez Morales et al., 1996). In mice, experimental infections in nude (lacking T cells) and severe combined immunodeficiency (SCID) (lacking T and B cells) mice are chronic and lethal (Heine et al., 1984; Mead et al., 1991). In particular, CD4^+^ lymphocytes are crucial for the resolution of infection; mice depleted with anti-CD4^+^ antibody have markedly decreased immunity (Chen et al., 1993a). Adoptive transfer of CD4^+^ IELs (nonconventional lymphocytes located among the epithelial cells in the lumen) cells in SCID mice reduces parasite load and confers better protection against *Cryptosporidium* infections than CD8^+^ IELs (McDonald et al., 1996). CD8^+^ T-cells play an important though probably lesser role as these cells secrete IFN-γ early in infection (Leav et al., 2005) and may reduce the parasite load in the infected intestine *via* cytotoxicity by lysing infected intestinal epithelial cells (Pantenburg et al., 2010).

Antibodies also play a role in immune response as individuals with deficiencies such as X-linked hyper-IgM and IgA deficiencies are more susceptible to *Cryptosporidium* infection (Stiehm et al., 1986; Winkelstein et al., 2003; Wolska-Kusnierz et al., 2007). However, mice that lack B cells are able to clear infection (Chen et al., 2003). Following primary infections, antibody responses, specifically IgG and IgA, are mounted against the parasite (Ungar et al., 1986; Kassa et al., 1991). In the general population, the seropositivity rate in humans is high; reported to be anywhere from 25 to >60% depending on the location and population being surveyed (Priest et al., 2001; Frost et al., 2004). Antibodies provide some protection from reinfection (Fayer et al., 1990) and are directed against several immunodominant antigens that block parasite invasion *in vitro* or reduce parasite loads in mice (Arrowood et al., 1989; Riggs et al., 1989; Bjorneby et al., 1990; Petersen et al., 1992; Joe et al., 1998; Jenkins et al., 1999).

While exposure rates in North America are high, as indicated by high seropositivity rates (Ong et al., 2005), the parasite has a global distribution. It is second only to rotavirus as a cause of childhood diarrhea where it may result in cognitive impairment and growth deficits (Kotloff et al., 2013). The only FDA-approved anticryptosporidial drug is nitazoxanide, but it is not efficacious in immunocompromised patients (Abubakar et al., 2007). Additionally, there are no vaccines for cryptosporidiosis available. While adaptive immunity is critical for clearing the parasite completely, innate responses are important in reducing the number of parasites and in the initial stages. This review will highlight the host cell and early immune responses in the early stages that are important in controlling *Cryptosporidium* infections.

## *Cryptosporidium* infection and initial responses of the host cell

Cryptosporidial infections are usually initiated through ingestion of oocysts *via* contaminated food or water. Oocysts pass through the stomach to the small intestine where they excyst and release sporozoites. Sporozoites then invade the brush border of epithelial cells and subsequently differentiate into trophozoites (growing stage). Like other apicomplexan parasites, including *Plasmodium* and *Toxoplasma*, *Cryptosporidium* has a complex life cycle producing asexual and sexual stages, ultimately resulting in oocysts shed in the feces which are fully infectious upon leaving the host (Current and Reese, 1986).

The parasite occupies an unique niche in the epithelial cells in that the parasite is enclosed in a parasitophorous vacuole of both parasite and host cell origin. This location in the cell is described as “intracellular but extracytoplasmic” as the parasite resides inside the cell, i.e. beneath the host cell membrane. The base of the parasitophorous vacuole is adjacent to the host cell cytoplasm and a complex “feeder organelle” is located at the interface, presumably to facilitate transport into and out of the host cell cytoplasm. The parasitophorous vacuole has an apical surface facing the gastrointestinal (GI) lumen. Intestinal epithelial cells (IECs) and mucosa in the gut form a physical protective barrier against many pathogens and also serve as an interface between the lumen and underlying immune system. Injury to these barriers due to infection and inflammation may result in increases in the uptake of solutes and microbial antigens (Griffiths et al., 1994) or may alter tight junctions between the epithelial cells as levels of the tight junction proteins occludin and claudin are reportedly decreased following *C. parvum* infection of Caco-2 cells (Kumar et al., 2018). In addition to increased permeability of the epithelial barrier, induction of pro-inflammatory responses occurs. One main major path is through the inflammation transcription factor nuclear factor kappaB (NF-κB) (Chen et al., 2001).

**FIGURE 1:**
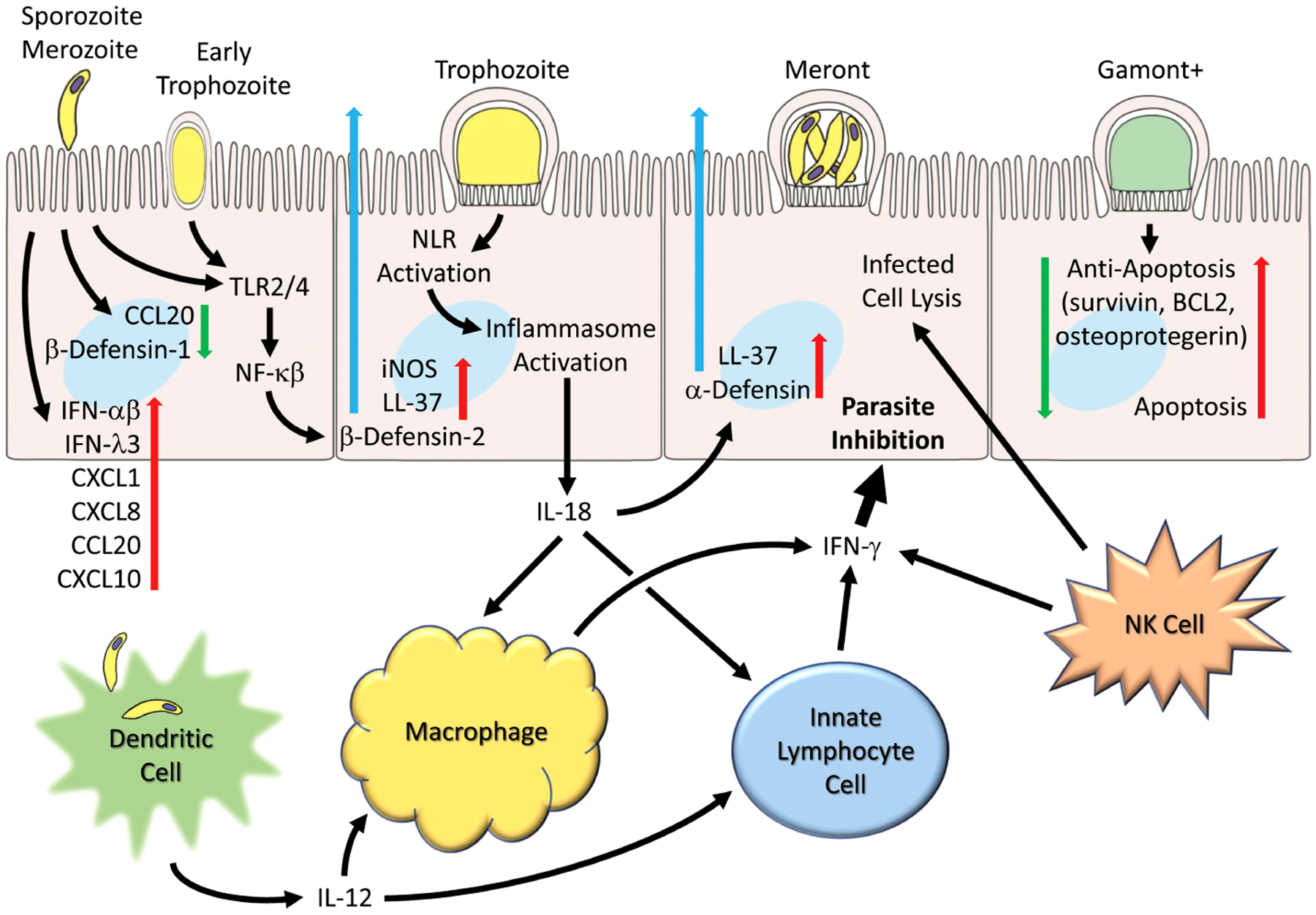
Generalized schematic illustrating early and host cell responses to *Cryptosporidium* infection. Parasite life cycle stage progression (left to right) following invasion by sporozoites (and merozoites) are shown as representative and the schematic pathways are not necessarily restricted to particular stages. Parasite infection leads to suppression of certain chemokines (e.g. CCL2) and β-defensin-1. Toll receptors (TLR2 and TLR4) are activated and lead to production and secretion of antimicrobial peptides (e.g. LL-37 and β-defensin-2). Inflammasome activation occurs and leads to IL-18 production. Numerous chemokines (e.g. CCL2, CXCL1, CXCL8, CXCL10), interferons (IFN-α, IFN-β and IFN-λ) and cytokines are released. Infection by *C. parvum* induces apoptosis but is countered by induction of anti-apoptotic genes (survivin, BCL2, and osteoprotegerin). Dendritic cells produce IL-12 in response to parasites or parasitic antigen and together with IL-18 released from epithelial cells helps to activate macrophages that generate IFN-γ. Innate lymphocyte cells (ILC1s) and NK cells also generate IFN-γ which can act on epithelial cells to inhibit the parasite.

Upon encountering microorganisms, intestinal epithelial cells are able to detect pathogens through innate receptors and respond by generating cytokines and upregulate chemokines which attract and activate other immune cells (Figure 1). *C. parvum* infection is reported to upregulate several chemokines including CXCL1 and CXCL8 (Laurent et al., 1997) that attracts neutrophils, CCL2 that attracts monocytes (Auray et al., 2007), CXCL10 that attracts dendritic cells (Lantier et al., 2013), CX3CL1 in biliary cells (Zhou et al., 2013) and CCL20 that have direct cytolytic activity against the free parasite (Guesdon et al., 2015). Additionally, adhesion molecules, such as beta-7 integrin (Mancassola et al., 2004) and ICAM-1 (Gong et al., 2011) that attract and activate other immune cells are up-regulated in *C. parvum* infection. Histocompatibility complex (MHC) class I and class II molecules are upregulated upon infection (Gomez Morales et al., 1995) and activation of Toll-like receptor (TLR) molecules have been reported in response to cryptosporidial infection (Chen et al., 2001; Chen et al., 2005). Nitric oxide produced through the induction of nitric oxide synthase (iNOS) of epithelial cells is significantly increased in *C. parvum* infection (Leitch and He, 1999; Gookin et al., 2004) and may be enhanced through dietary supplementation of L-arginine as demonstrated in several animals models (Leitch and He, 1994; Gookin et al., 2004; Castro et al., 2012). iNOS expression in *C. parvum* infection gives rise to increases in prostaglandin 2 (PGE2) synthesis which may maintain barrier function and promote clearance of infected epithelial cells (Gookin et al., 2004).

## Toll receptors

Toll receptors (TLR), molecules on the surface of epithelial and other immune cells that recognize broad patterns of microbial molecules, are constitutively expressed on IECs and are important activators of innate immunity. Not surprisingly, they have been found to play a role in host responses to *Cryptosporidium* infection. Recognition of microbial proteins through toll receptors leads to the activation of the adaptor protein myeloid differentiation protein (MyD88) required for most TLR signaling. Mice lacking MyD88 are more susceptible to infection compared to wild-type mice and more so if the mice are made immunodeficient by IFN-γ neutralization (Rogers et al., 2006). Additionally, in human biliary epithelial cells, infections *in vitro* result in recruitment of TLR2 and TLR4 and are mediated by the activation of NF-κB activation, and result in the production of human β-defensin (Chen et al., 2005). When siRNA was used to knock down the expression of TLR2, TLR4, or MyD88, *C. parvum*-induced NF-κB activation was blocked, indicating the importance of Toll receptor pathways in defense (Chen et al., 2005). In a biliary model of cryptosporidiosis, infections in TLR4 deficient mice were more severe and unresolving compared to controls (O’Hara et al., 2011). Likewise, in a model of malnourished mice, increased susceptibility correlated to decreased expression of TLR2 and TLR4 in the intestinal tract (Costa et al., 2011). However, TLRs may be less prominent or less responsive in the intestinal environment or may be “model dependent” as deficiencies in TLR2 and TRL4 did not increase infection in a neonatal mouse model (Lantier et al., 2014).

TLR4 is up-regulated in response to *C. parvum* infection in infected cholangiocytes and is regulated by the microRNA, let7i. Suppression of the microRNA occurs soon after infection, resulting in the increased expression of TLR4 (Chen et al., 2007). Activation of TLR4 also promotes the release into the lumen of exosomes which contain antimicrobial peptides which can bind to the surface of sporozoites (Hu et al., 2013). The toll receptor TLR9 may also play some role as administration of the ligand CpG (which acts through TLR9) reduced infections in neonatal mice by 95% and increased IL-12 and IFN-γ compared to controls (Barrier et al., 2006). However, CpG was found to be less effective in an adult model of malnutrition (Costa et al., 2011). Toll receptors certainly play an important role in dendritic cell response to *C. parvum* infection, as dendritic cells from MyD88 knockout mice are unable to generate cytokines such as IL-12 in response to incubation with live sporozoites or antigen (Bedi and Mead, 2012).

## NOD-like receptors and Inflammasomes

Pathogens are also sensed intracellularly in the cytoplasm by NOD-like receptors (NLRs) which can activate immune defenses through inflammasome formation and may lead to the cleavage and activation of the proinflammatory cytokines IL-1β and IL-18. The latter cytokine, IL-18, is increased in infected mice (Ehigiator et al., 2005) and infected HCT-8 cells (McDonald et al., 2006). Additionally, IL-18 knockout mice are more susceptible to infection (Ehigiator et al., 2007).

The processing and secretion of mature IL-18 (and IL-1β) is mediated by caspase-1 which is activated within an inflammasome following the engagement of inflammasome-initiating sensors. In a report by McNair et al. (McNair et al., 2018), it was found that Casp-1^−/−^/Casp-11^−/−^ knockout mice are over 35 times more susceptible to *C. parvum* infection than wild-type mice. Susceptibility correlated with a lack of IL-18 in caspase-1 and caspase1/11 knockout mice, as IL-18 is significantly elevated in wild-type mice. IL-1β was not generated in any significant amount following infection nor was any increased susceptibility observed in IL-1β nor IL-1βR knockout mice. These observations were corroborated in a recent study using a *C. tyzzeri* mouse model (Sateriale et al., 2021). This study further extended the previous work to show that mice lacking NLRP6, (an NLR known to induce IL-18 in the gut) were more susceptible to infection and had decreased levels of IL-18. Epithelial cells were shown to be the predominant cells generating IL-18. In contrast, mice lacking other NLRs such as NLRP3, NLRP1b, Aim2 and NLRC4 (which tend to induce an IL-1 response) were no more susceptible to infection than wild-type mice (McNair et al., 2018; Sateriale et al., 2021). It was also shown that the adapter protein ASC is important to susceptibility, and that the caspase-1 canonical inflammasome signaling pathway is the dominant pathway in *C. parvum* resistance (McNair et al., 2018) compared to the non-canonical pathway/caspase pathway activated in other pathogens like *Leishmania* (de Carvalho et al., 2019).

## Interferons

Type I interferons are mainly thought of as an innate defense mechanism to viral infections. However, many protozoan infections induce interferon α/β (IFN-α/β) in response to infection, including *Cryptosporidium*. *In vitro*, CMT-93 cells expressed IFN- α/β in response to *C. parvum* infection. Type I interferons were found to be protective as treatment with IFN-α or IFN-β before infection reduced parasite numbers significantly. In the same study, bonemarrow derived dendritic cells were also shown to generate IFN-α/β in response to live parasite (Barakat et al., 2009b). *In vivo*, Type I IFNs were expressed in the intestinal tissue of neonatal SCID mice 24 h after infection, while treatment with anti-IFN-α/β-neutralizing antibodies increased infection (Barakat et al., 2009b). The long non-coding RNA (NR_033736) is involved in the regulation of type I interferons and is increased in intestinal epithelial cells following *C. parvum* infection (Li et al., 2021).

Type III interferons, or IFN-λs, are important mediators of viral infections (Nice et al., 2018) and bacterial infections as well (Odendall et al., 2017). IFN-λs are particularly important in mucosal infections because of their preferential expression of the IFNLR1 receptor on epithelial cells. In a report by Ferguson et al., these genes were found to be upregulated in *C. parvum*-infected porcine epithelial cells (Ferguson et al., 2019). Monolayers pretreated with IFN-λ protected cells, resulting in significant reduction of infection by limiting invasion and decreasing maturation of parasites. Also, IFN-λ offset the loss of barrier function due to infection by decreasing paracellular permeability due to sodium. Administration of neutralizing antibodies against IFN-λ to neonatal mice increased *C. parvum* infection during peak infection. In another study, using a genetic screen (CRISPR-cas9 knockout) to identify human proteins involved in survival, it was found that infection of host cells resulted in an increase of predominately type III interferons compared to type I (Gibson et al., 2022). Additionally, production of type III interferons was shown to be dependent on TLR3 signaling as mice lacking TLR3 were more susceptible to infection and had significantly reduced levels of IFN-λ.

## Antimicrobial peptides

Other immune effectors generated in the epithelial cell in response to infection include antimicrobial peptides. In mammals, there are two large families of antimicrobial peptides: defensins and cathelicidins. The expression of β-defensins, which are widely expressed in epithelial cells, was studied using a *Cryptosporidium*-infected human colonic HT29 cell line (Zaalouk et al., 2004). Infection of these cells induced the expression of human β-defensin-1 (hBD-1), a partial reduction in the expression of human β-defensin-2 (hBD-2), but had no effect on human β-defensin-3 (hBD-3) gene expression. Antimicrobial peptides such as LL-37 (human) or murine cathelicidin related antimicrobial peptides (CRAMP) and β-defensins can result in the lysis of the parasitic cell membrane. Antimicrobial peptides have been found to lyse sporozoites *in vitro* (Arrowood et al., 1991; Carryn et al., 2012; McDonald et al., 2013). Exogenous administration of CRAMP decreased *C. parvum* infection in neonatal mice (Guesdon et al., 2020). Typically, CRAMP expression is higher in neonatal mice during the first 3 weeks of age to help protect neonatal mice from a number of intestinal infections. However, CRAMP expression was found to be reduced during *C. parvum* infection, while other antimicrobial peptides were upregulated (Guesdon et al., 2020), suggesting the possibility that the parasite may have a mechanism for downregulating this mediator. These antimicrobial peptides can be released through exosomes from epithelial cells (Hu et al., 2013). The activation of these exosomes is initiated through the TLR4 signaling pathway that downregulates microRNA Let-7, which in turn results in an increase of protein SNAP23 involved in vesicular exocytosis. Interestingly, other effector molecules (epithelial cell-derived proteins and cryptosporidial RNAs) have been shown to be released *via* exosomes and shuttle to the spleen, activating immune cells distal from the site of infection (Wang et al., 2019b).

## Complement and mannose binding protein

*C. parvum* can activate both the classical and lectin complement pathways, leading to the deposition of C3b on the parasite (Petry et al., 2008). However, increased susceptibility to the parasite could only be demonstrated in adult mice lacking mannose binding lectin (MBL) not in mice lacking C1q or in wild-type mice, indicating the importance of the lectin pathway and suggesting the alternative pathway does not play a major role in infection. MBL is a conserved protein that binds to microbial surfaces and promotes opsonophagocytosis. Studies have suggested the protective role of MBL may be most important in young children and individuals with immunodeficiencies (Kelly et al., 2000; Kirkpatrick et al., 2006). In 5% of the world’s population, polymorphisms in the *MBL2* gene create low MBL-producing *MBL2* genotypes, which may lead to an increased susceptibility to particular diseases, including cryptosporidiosis (Carmolli et al., 2009). In a recent study involving children in South India under the age of 3, polymorphism in the MBL2 gene was associated with susceptibility to cryptosporidial diarrhea (Liakath et al., 2021).

## IFN-γ and other key cytokines

In general, intracellular pathogens induce a strong cell-mediated (Th1) response where IFN-γ plays a leading role in driving the cellular response and also in effector mechanisms inhibiting the parasite. IFN-γ is a key cytokine important in both human cryptosporidiosis and experimental animal studies (McDonald and Bancroft, 1994; Hayward et al., 2000). Depletion of the IFN-γ gene in mice or neutralization using anti-IFN-γ antibodies results in greater susceptibility to infection (Theodos et al., 1997; Griffiths et al., 1998; Mead and You, 1998). Sources of IFN-γ include NK cells, macrophages, and T cells. An early source of IFN-γ, produced soon after infection, are intestinal intraepithelial lymphocytes (iLEL), particularly CD8αTCRα cells which were found to generate IFN-γ as soon as 24 hours post infection (Leav et al., 2005). Most recently, innate lymphocyte cells (ILC1s) have been identified as a major source of IFN-γ (Gullicksrud et al., 2022).

While IFN-γ importance in infection is well established, the mechanism by which it aids resistance to infection is not fully understood. IFN-γ has a direct effect on infected epithelial cells resulting in decreased infection (Pollok et al., 2001). It has been suggested that this is due, in part, to depletion of available iron (Fe) in the cell as Fe^+^ supplementation in culture media can partially reverse the inhibition of parasite growth (Pollok et al., 2001). Transcriptional profiling of epithelial cells from infected mice has shown that an upregulation of IFN-γ induced the effectors indoleamine 2,3-dioxygenase (IDO), guanylate binding protein (GBP) and immunity-related GTPases (IRG) (Gullicksrud et al., 2022). Additionally, while deletion in IDO and GBP did not increase susceptibility to *C. parvum* infection, mice lacking the GTPases, immunity-related GTPase family M (IRGM)1 and IRGM3 were more susceptible to infection compared to wild-type mice. In another study looking at changes in m^6^A mRNA methylation (which may regulate innate immune responses), expression levels of IRGM2 and IFN-γ induced GTPase IGTP or IRGM3 were found to be increased in infected cells (Xia et al., 2021).

Two key inducers of IFN-γ, IL-12 and IL-18, are important in primary immune responses to infection. Both cytokines work synergistically to produce IFN-γ (Choudhry et al., 2012). IL-12 was shown to be protective in mice (Urban et al., 1996) while lack of this cytokine leads to greater susceptibility and severity of infection (Ehigiator et al., 2003). Dendritic cells can generate IL-12 in response to *C. parvum* (Bedi and Mead, 2012). Specially, batf3-dependent CD103^+^ has been shown to be a source of IL-12 in neonatal mice (Potiron et al., 2019). IL-12 synergizes with IL-18 to stimulate effector cells (ILC1s) to produce IFN-γ (Gullicksrud et al., 2022). Additionally, IL-4 has also been shown to work synergistically with IFN-γ to enhance parasite killing (Lean et al., 2003).

TNF-α, a cytokine that is increased during infection, does reduce parasite numbers in infected epithelial cells *in vitro* through limiting invasion of host cells (Barakat et al., 2009b). However, the role for TNF-α *in vivo* may be less apparent as mice treated with anti-TNF-α or TNF-α deficient mice are no more susceptible than control mice (Chen et al., 1993b; Lean et al., 2006). However, in the absence of IFN-γ, protection was observed in IFN-γ knock out mice treated with TNF-α (Lacroix et al., 2001).

IL-10 and TGFβ are key regulators in the maintenance of immunological responses in the gastrointestinal tract. Both of these cytokines are increased during the resolution of cryptosporidial infections (Robinson et al., 2000; Lacroix et al., 2001). In particular, TGFβ has an inhibitory effect on IFN-γ (Lean et al., 2003).

## Microbiome and metabolites

The microbiome has become an important focus in the interactions of gut infection susceptibility and host response. Since *Cryptosporidium* infections are primarily localized to the epithelium of the intestine it is reasonable to assume that the local flora would impact cryptosporidial infection and the over-all disease state. In early studies, SCID mice with established conventional gut flora showed more resistance to infection by *C. parvum* than germ-free SCID mice in that oocyst production was delayed by several weeks (Harp et al., 1992). A TCR-α-deficient mouse model of cryptosporidiosis reportedly developed IBD-like lesions following infection (Sacco et al., 1998). Interestingly, TCR-α-deficient mice exhibited more severe mucosal lesions among infected germ-free versus mice bearing conventional gut flora (Sacco et al., 1998), suggesting microbiome influences on the pathology of disease. Additionally, gut flora may play a protective role through the activation of the TLR5-MyD88 pathway, stimulating dendritic cells and helping to generate cytokines, such as IL-12, and type I and type II IFNs (Lantier et al., 2014).

Differences in microbiome composition may correlate with differences in susceptibility and clinical symptoms. In one human study, stools from *C. parvum* infected volunteers showed changes in microbiota that correlated with differences in susceptibility to infection and also to shifts in metabolites (Chappell et al., 2016). In particular, fecal indole or indole producing bacteria were associated with protection. In another study, when comparing 72 infants with and without diarrheal symptoms, low *Megasphaera* abundance was associated with diarrheal symptoms prior to and at the time of *Cryptosporidium* detection (Carey et al., 2021). Interestingly, *Megasphaera* are known to synthesize short-chain fatty acids which help maintain the homeostasis of the intestinal tract.

Infections with *C. parvum* can also alter the microbiome (Ras et al., 2015). In animals, cryptosporidial infections in goat kids depleted butyrate-producing bacteria (Mammeri et al., 2020), changes in infected neonatal mice showed increased proportions of the bacterial phylum Bacteroidetes (Mammeri et al., 2020). Monkeys with active infection were found to have enriched taxa associated with dysbiosis, inflammation and rapid transit time thorough the gut (McKenney et al., 2017). Shifts in fungal microbiome in horses have also been observed after cryptosporidial infections (Wang et al., 2022).

Additionally, drugs and natural products may also alter the gut flora and influence infection (Gorla et al., 2014; Rahman et al., 2022). In a mouse model of cryptosporidiosis, increased severity of infection in antibiotic-treated mice depleted of primary Gram positive bacteria was associated with a decrease in short chain fatty acids (Charania et al., 2020). In animals, prebiotics (diet without fiber) altered the microbiome in *C. parvum* infected mice and enhanced infection, (Oliveira et al., 2019) whereas probiotics often used to bolster the microbiome were also found to increase infection (Oliveira and Widmer, 2018). Limited studies have been performed in humans. In one study, a probiotic was associated with enhanced resolution of disease (Pickerd and Tuthill, 2004) while another study showed improvement in barrier permeability among children infected with *Cryptosporidium* who were given *Lactobacillus* (Sindhu et al., 2014).

Bacteria produce many metabolites which can affect immune populations, such as T regulatory cells, or have a direct effect on epithelial cells themselves. In a study examining changes in flora and metabolites in neonatal mice, medium or long chain saturated fatty acids inhibited *C. parvum* growth while long-chain unsaturated fatty acids enhanced *C. parvum* invasion (VanDussen et al., 2020). Short chain fatty acids such as butyrate and propionate (produced mainly by Gram positive bacteria and depleted with certain antibiotics) were also found to have a direct inhibitory effect on *C. parvum* growth *in vitro* (Keelaghan et al., 2022). In another study looking at microbiome and metabolite changes during infection, bacteria such as Coriobacteriaceae and *Lactobacillus* were increased along with Damino acids and short chain fatty acids while glycolysis/citrate cycle metabolites were depleted (Karpe et al., 2021).

## Apoptosis and autophagy

Apoptosis is a form of programmed cell death that helps in the elimination of pathogens and abnormal cells. Apoptosis is initiated through either an intrinsic pathway (from within the cell) and or an extrinsic pathway (signals from other cells). Both of these pathways involve activating caspases which result in degrading proteins. There is an apparent interaction between the host and parasite genes with the host trying to eliminate the parasite through apoptosis and parasite induced suppression of some host apoptotic genes allowing for increased growth of the organism. Initially, apoptosis is up-regulated for the first few hours during invasion, perhaps as a way to dampen inflammatory responses (Mele et al., 2004). Anti-apoptotic genes are then up-regulated during development of the trophozoites at 24 hours (Mele et al., 2004). Inhibiting apoptosis occurs as a way to counter host cell response and to garner host metabolites in a dividing cell. These genes include BCL-2, osteoprotegerin and survivin (Mele et al., 2004; Castellanos-Gonzalez et al., 2008; Liu et al., 2008). When the latter was knocked down, an increase in apoptosis was observed which resulted in a decrease in parasite growth (Liu et al., 2008). At the same time (24 hours post infection), a moderate amount of apoptosis still occurs (McCole et al, 2000), reported mainly in noninfected adjacent cells (Chen et al., 2001; Mele et al., 2004) perhaps as a way to limit parasite expansion by the host. Induction of apoptosis occurs in the later stages of infection (48 hours post infection) perhaps as a way to facilitate escape by the parasite from the host (Chen et al., 1999; McCole et al., 2000; Buret et al., 2003). Alternatively, host-cell apoptosis may be advantageous for the host and a way to eliminate infected cells but in a controlled manner. In *exvivo* enterocytes from piglets, repression of apoptosis by the host cell has been reported to occur except at the villus tips allowing for elimination of infected cells in a more controlled manner while still maintaining integrity of the epithelial barrier (Foster et al., 2012). Studies with microRNA show that many different microRNAs are involved in apoptosis and anti-apoptosis pathways (Wang et al., 2019a). One microRNA, miR-942–5p, which is up-regulated in HCT-8 cells following activation of the TLR2/TLR4-NF-κB signaling pathway by *C. parvum*, has been implicated in regulation of host cell apoptosis (Zhang et al., 2020) through the TRAIL-dependent pathways (Xie et al., 2022).

Another mechanism through which host cells help eliminate intracellular pathogens is through the process of autophagy. In order to maintain homeostasis, the host cell recycles degraded macromolecules and organelles. Alternatively, autophagy processes may be used to target intracellular pathogens by autophagosome formation around the free pathogen or pathogen within a vacuole, fusing with lysosomes and ultimately the degradation of the pathogen. There is also some evidence to suggest that immunity-related GTPases are linked to the autophagosome and may aid in disrupting pathogen vacuoles (Evans et al., 2018). In a recent study, autophagy was induced in *C. parvum*-infected CaCo2 cells. This was demonstrated through increased LC3II/I ratio (increase in this ratio suggests autophagy induction) and enhanced autophagosome formation (Priyamvada et al., 2021). While some intracellular protozoa such as *T. gondii* and *T. cruzi* have mechanisms to interfere with the autophagy process of the host cell (Muniz-Feliciano et al., 2013; Portillo et al., 2017; Onizuka et al., 2017), this has not yet been shown for *C. parvum*. However, silencing of the autophagy ATG7 gene results in decreased expressing of two barrier proteins, occludin and claudin, suggested that there may be a link between *C. parvum* induced autophagy and barrier integrity (Priyamvada et al., 2021). Interestingly, in humans, single nucleotide polymorphisms (SNP) in the gene ATG16L1 impairs autophagy. SNPs in the ATG16L1 cause greater survival of intracellular pathogens (Evans et al., 2018). EL-Refai et al. were able to show that patients that had this SNP had greater severity of disease and increased risk of infection to *C. parvum* (El-Refai et al., 2021).

## Neutrophils

Neutrophils are important first responders to active infection sites, especially for bacteria but also for other pathogens as well. They provide important defense mechanisms such as phagocytosis and the production of reactive oxygen reactive species. Their role, however, in cryptosporidiosis may be less direct and more in augmentation of other cell populations. In cryptosporidiosis, neutrophils are recruited in the lamina propria soon after infection (Genta et al., 1993; Zadrozny et al., 2006). In piglets, depletion of neutrophils using an anti-CD18 antibody did not alter lipid peroxidation or peroxynitrite formation, suggesting these cells were not a significant source of free radicals nor did they have an influence on the severity or pathology of infection (Zadrozny et al., 2006). However, they were found to enhance barrier function (Zadrozny et al., 2006). SCID-beige mice lacking either macrophages or neutrophils have more severe infections when infected with *C. parvum* (Takeuchi et al., 2008). Adoptive transfer of either macrophages or neutrophils alone into these mice could not rescue the mice from succumbing to the infection but rather required both cell types. It was suggested that there may be a cooperative interaction been the two cell types (Takeuchi et al., 2008). In the same study, polymorphonuclear leukocyte cells (PMN)s isolated from *C. parvum*-infected mice were shown to play a role in converting resident macrophages to M1 macrophages. Another possible mechanism is through NETosis, an effector mechanism by which extracellular fibers (primarily DNA) from neutrophils bind pathogens (Hasheminasab et al., 2022). In one study, neutrophil extracellular traps (NETs) were found to form when bovine neutrophils were incubated with either sporozoites or oocysts. This led to the entrapment of 15% of the sporozoites (Munoz-Caro et al., 2015). PMNs exposed to sporozoites had increased levels of the chemokine CXCL8, TNF-α, IL-6, and the growth factor GM-CSF.

## Macrophages

Macrophages are a source of IFN-γ and contribute to clearance of cryptosporidiosis through phagocytosis of the parasite. Intact and partially digested parasites have been found in macrophages associated with M cells that overlay Peyer’s patches in guinea pigs (Marcial and Madara, 1986). Mice depleted of macrophages using clodronate-containing liposomes showed higher levels of infection than controls and were unable to mount a significant IFN-γ response suggesting macrophages are an important source of this cytokine (Choudhry et al., 2012). In neonatal mice, inflammatory Ly6C^+^ monocytes recruited into the subepithelial intestine and macrophages are shifted to a proinflammatory phenotype during infection, produce TNF-α and IL-1β. These cytokines, while enhancing other immune functions, also contribute to decreasing barrier function and increasing gut permeability (de Sablet et al., 2016). While macrophages generate IFN-γ and other immune factors they also require appropriate signals to be stimulated. Macrophages can be activated *in vitro* in the presence of *C. parvum* but require a combination of IL-18 and IL12 to generate significant amounts of IFN-γ as neither of these cytokines alone result in activation (Choudhry et al., 2012).

## Dendritic cells

Dendritic cells are important in the early stages of *Cryptosporidium* infection, as mice depleted of these cells are more susceptible to infection (Lantier et al., 2013; Bedi et al., 2014). They are recruited in the ileum of *C. parvum*-infected mice (Auray et al., 2007) and express important mediators such as IFN-α and IFN-β (Barakat et al., 2009b) and IL-12, IL-1β and IL-6 (Bedi and Mead, 2012) in response to live sporozoites or soluble sporozoite antigen. The former (live sporozoites) generated a better response as parasite burden was reduced with longer protection observed in mice adoptively transferred with dendritic cells stimulated with live sporozoites (Bedi et al., 2014). DCs capture parasites or phagocytized infected epithelial cells and transport them to the mesenteric lymph nodes (Ponnuraj and Hayward, 2001; Auray et al., 2007; Perez-Cordon et al., 2014). Gut flora may help in synergizing responses of DCs (through TLR5 signaling) as expression of IFN-α and IFN-β, as induced by poly(I:C), increased in the presence of commensal bacteria (Lantier et al., 2014). Additionally, they aid in the initiation of adaptive responses and activation of T cells, resulting in IFN-γ production (Bedi and Mead, 2012). In a study that examined different subsets of dendritic cells, CD103^+^ were found to control infections better than CD103^−^ DCs (Potiron et al., 2019). Since neonatal mice have fewer CD103^+^ cells, it has been suggested that this is one reason neonates are more susceptible to *C. parvum* infection (Potiron et al., 2019). Additionally, neonatal mice that lacked transcription factor Batf3 (Batf3 drives the development of CD103^+^ DCs) were more susceptible to infection and could not clear infection. These mice had a lower ability to generate IL-12 and were presumably not able to contribute to IFN-γ production. Lastly, mice deficient in cDC1 dendritic cells were found to be more susceptible to *C. Tyzzeri* infections. Infections in these mice generated skewed T cell responses that were predominately Th17 and Treg responses compared to IFN-γ -producing Th1 response generated by wild-type mice (Russler-Germain et al., 2021) demonstrating the importance of these cells in initiating the appropriate Th1 response.

## NK cells

NK cells contribute to innate immunity to cryptosporidiosis through lysis of infected epithelial cells or through production of IFN-γ. In neonatal lambs, NK cells (CR1^+^/CD16^+^ and NCR1^+^/CD16-populations) increase in response to infection (Olsen et al., 2015). They are activated by the cytokine IL-15 and have cytolytic activity against *C. parvum*-infected epithelial cells *in vitro* (Dann et al., 2005). Susceptibility to disease and severity of disease does increase in mouse models deficient in NK cells. Infections in beige mice (lacking NK cells) were slightly more susceptible (detectable by microscopy) than other strains of wild-type mice (Enriquez and Sterling, 1991). Infections in NIH III mice (SCID mice deficient in NK cells) were found to be more disseminated than SCID mice with normal NK cell function (Mead et al., 1991) and infections were more severe in Rag2^−/−^ gammaC^−/−^ mice (T and B cells, lack NK cells) than Rag2^−/−^mice (Barakat et al., 2009a). However, in experimental mouse models, depletion of NK cells using α-asialo-GM1 antibody against NK cells did not alter susceptibility or severity of infection (Rohlman et al., 1993; McDonald and Bancroft, 1994; McDonald et al., 2013), while depletion using anti-NK1.1 antibody did increase severity (Korbel et al., 2011), perhaps because the α-asialo-GM1 antibody does not completely deplete all target cells, including tissue NK cells (Victorino et al., 2015). In a recent study, Rag2^−/−^ mice were treated with α-asialo-GM1 which, once again, was found to deplete NK spleen cells but did not deplete innate lymphoid cells (ILCs), a type of NK cell that resides in the gut mucosal (Gullicksrud et al., 2022). Additionally, mice that lack T, B and splenic NK cells remained more resistant, with less severe infections, compared to *Rag2*^−/−^/*Il2rg*^−/−^ that lacked all 4 cell populations (T, B, NK and ILC cells). Innate lymphoid cell type 1s (ILC1s) were found to be protective in the early stages of cryptosporidiosis and an important source of IFN-γ (Gullicksrud et al., 2022).

## Immune evasion

*Cryptosporidium parvum* has developed strategies to evade or alter the host cell response. The parasite’s unique location (intracellular but surrounded by host cell membrane) may play a role in evasion. While the intracellular location may help in avoiding exposure to immune effector mechanisms such as antibodies and phagocytosis, the extracytoplasmic location avoids host cellular defense mechanisms such as Guanylate Binding Proteins (GBP)s that interact with vacuoles that are completely intracellular. This unique location may also limit exposure of parasite antigens to immune cells in the lamina propria.

Another potential mechanism of immune evasion involves inhibiting signal transduction pathways in the host cell. STAT-1α is a major component of IFN-γ signaling and important in the immune response as mice with STAT-1α knocked out are more susceptible to infection (Ehigiator et al., 2007). *C. parvum* infection in CMT-93 cells was shown to decrease several IFN-γ-dependent genes, including indoleamine 2,3-dioxygenase (IDO) which catabolizes tryptophan to kynurenine (Choudhry et al., 2009). Investigating possible mechanisms, it was found that *C. parvum* infection caused depletion of the STAT-1α protein in infected host cells, leading to the down-regulation of IFN-γ (Choudhry et al., 2009). Likewise, p38 MAPK signaling is downregulated (in particular Mk2 and Mk3) in infected cells (He et al., 2021). MAP kinase-activated protein kinases are important for a number of immune responses.

As described above, *C. parvum* inhibits apoptosis early in infection allowing it to establish itself but pro-apoptotic gene expression appeared to be favored at late stages (Liu et al., 2009). The chemokine CCL20, a chemokine involved in dendritic cell recruitment, was found to be downregulated after *C. parvum* infection, independently of an IFN-γ response or the presence of microbiota (Guesdon et al., 2015). Additionally, *C. parvum* has been shown to deliver transcripts into the nucleus of host cells, resulting in the modulation of a number of host genes (Wang et al., 2017). In particular, delivery of the parasite RNA transcript, cdg7_FLc_1000 to the host cell down regulates the β-defensin-1 gene, contributing to evasion early in infection (Ming et al., 2018).

## Conclusion and future directions

In the recent years, much research has focused on host cell responses and innate immunity to cryptosporidiosis. Advances have been made in understanding how the control of those responses occurs which is often through nucleic acid molecules such as miRNA (Ming et al., 2017) and long non-coding (linc) RNA (Mathy et al., 2022) which help in the regulation of various immune mediators. Additionally, studies on less studied mucosal cell types such as innate lymphocytes will remain an area of interest. The microbiome and metabolites will continue to be a focus as it is discovered how these bacteria and other microbes affect host cell and parasite interactions. New *in vitro* models including 2D and 3D culture systems, organoid/enteroid (Zhang et al., 2016; DeCicco RePass et al., 2017; Heo et al., 2018) and stem cell cultures (Wilke et al., 2019) will facilitate extended or continuous culture of the parasite and support the study of hard to propagate strains such as *C. hominis*. Cell culture models of greater complexity and control may make it possible to study interactions of individual cell types of the immune systems with epithelial cells and other components (e.g. microbiome, metabolites of the intestinal environment) in an *in vitro* setting.
